# Changes in body composition and average daily energy expenditure of men and women during arduous extended polar travel

**DOI:** 10.1371/journal.pone.0308804

**Published:** 2024-10-10

**Authors:** Adrian J. Wilson, Robert M. Gifford, Henry Crosby, Sarah Davey, Natalie Taylor, Mike Eager, C. Doug Thake, Christopher H. E. Imray

**Affiliations:** 1 Department of Research and Development, University Hospitals Coventry and Warwickshire NHS Trust, Coventry, United Kingdom; 2 Department of Physics, University of Warwick, Coventry, United Kingdom; 3 Academic Department of Military Medicine, Research and Clinical Innovation, Royal Centre for Defence Medicine, Birmingham, United Kingdom; 4 Centre for Cardiovascular Science, University of Edinburgh, Edinburgh, United Kingdom; 5 35 Engineer Regiment, Carver Barracks, Wimbish, Saffron Walden, Essex, United Kingdom; 6 Centre for Physical Activity, Sport and Exercise Sciences, Coventry, United Kingdom; 7 Academic Department of Military General Practice, Royal Centre for Defence Medicine, Birmingham, United Kingdom; 8 Department of Anaesthetics, St Mary’s Hospital, Imperial College Healthcare NHS Trust, London, United Kingdom; 9 Faculty of Medicine, Imperial College London, London, United Kingdom; 10 Department of Vascular and Renal Transplant Surgery, University Hospitals Coventry and Warwickshire NHS Trust, Coventry, United Kingdom; Università degli Studi di Milano: Universita degli Studi di Milano, ITALY

## Abstract

Weight and skin-fold measurements were made at five-day intervals during a 47-day expedition by six men and three women from the edge of the sea ice to the South Pole. From these, together with detailed manual records of the nutrition for individual participants, the average daily energy expenditure was determined before and after a resupply at approximately mid-point of the expedition. For all participants body weight fell during the expedition with the overall loss being much smaller for the three female participants (-4.0, -4.0, -4.4kg) than for the male participants, (mean±sd) -8.6±2.0kg. Fat weight fell approximately linearly during the expedition with a total loss of (-4.1, -6.5 and -2.5kg) for the three female participants and -6.8±1.7kg for the male participants. Individual fat-free weight changed by a smaller amount overall: (0.13, 2.5 and -1.8kg) for the three female participants; -1.8±2.0kg for the male participants who, with one exception, lost fat-free tissue All participants showed a substantial variation in fat-free tissue weight during the expedition. Analysis of the daily energy expenditure showed adequate nutrition but the intake fell for the second part of the expedition although the reasons for this are unclear, but adaptation to the cold, altitude and workload are possible explanations. The validity of this time-averaged measurement for individual participants was determined from analysing moments about the mean of time-series actigraphy data from wrist worn devices. The mean and autocorrelation function of the actigraphy data across subjects were analysed to determine whether measures could be compared between participants. The first, second and third moment about the mean of the day-to-day activity was found to be time-invariant for individual subjects (χ^2^, p>0.05) and the normalized mean and autocorrelation measured over a day for each participant indistinguishable from the mean of the group (χ^2^, p>0.05) allowing both longitudinal and cross-sectional analysis.

## Introduction

Matching calorific intake to energy expenditure for those undertaking extreme activities is essential if a substantial loss of body weight through negative energy availability (EA) is to be avoided and is a key component of achieving a successful endeavour. In addition, previous work on polar expeditionary journeys has shown minimal metabolic consequences post-expedition if adequate nutrition is achieved [1–3]. Matching energy expenditure to the calorific value of nutritional intake requires measuring the energy expenditure during extreme activities, which is challenging. Whilst indirect calorimetry has been used in studies where activities are focussed on one geographical location [4] this is not an appropriate technique for those undertaking expeditionary journeys in the polar environment, in the mountains or on the oceans. It has been argued that the Doubly Labelled Water (DLW) technique [5] is the only realistic way of measuring metabolic rate during expeditionary journeys [6]. In its standard form the technique gives the energy expenditure due to the carbohydrate and lipid substrate utilisation [5] and has been used in polar expeditions [7, 8], oceanic rowing [9], mountaineering expeditions [10], on astronauts at the International Space Station [11] and in arduous military training [12]. A modified version of the technique using ^15^N to measure protein metabolism [13] has been used in transantarctic expeditions [14]. It should be noted that where expeditions involve substantial geographic movement and usage of local water supplies, changes in the abundance of ^1^H isotopes with location give problems in establishing baseline levels of the isotopes [7, 8, 15]. Analysis procedures [7] and the use of control subjects [11] have been used to address this problem. In addition to these measurement limitations, there are logistic challenges in using DLW in that urine samples need to be collected, preserved and transported throughout the expedition, and then potentially transnationally, for analysis.

Using the conservation of energy (CoE), it is possible to calculate the total energy expenditure from the nutrition consumed and the changes in body composition [1, 2]. For this to be successful, accurate records need to be kept of the calorific value of the food consumed which is challenging in expeditionary environments where ‘graze bags’ allow participants to consume variable amounts of nutrition outside meal-times.

The CoE method essentially measures the total energy consumed during the expedition, from which the average daily energy expenditure is calculated whilst the DLW methods give average daily energy expenditure. However, both the CoE and the DLW methods are time-averaged measures that only give a meaningful reflection of the daily energy expenditure if the daily routine and activity are largely time invariant, analogous to data that is termed ‘stationary’ in classical random data time-series analysis [16].

There are two additional complications for the CoE method. Firstly, it is assumed that loss of body weight is linear during the expedition which is not seen in starvation studies [17] albeit the latter are normally over longer periods of time. Secondly that fat and fat-free tissue have different calorific values [e.g. 18] and there is the assumption that the loss of fat and fat-free tissue occur at the same rate during the expedition.

Recent studies using the CoE method to determine energy expenditure during expeditions [1, 2] have used weight and body composition measurements made in the UK, typically two to three weeks before and after the start and end of the expedition. This represents both a fundamental limitation and an opportunity for the method. The limitation is that in the period before the expedition starts and, in particular, after it has ended participants have access to uncontrolled calorific and macronutrient intake and so the weight and body composition will have changed by an unknowable amount in the intervening period. The opportunity is that laboratory-based measurements allow investigation of how body composition changes in different anatomical regions and thereby give the potential to better understand the physiological changes that occur in response to the environmental and workload challenges for participants undertaking unassisted expeditionary travel in polar regions. The environmental challenges include temperature down to -50°C and exposure altitudes up to 3,000m (FiO2 equivalent to approximately 14% at sea level) which is classed as ‘high altitude’ [19].

In this paper we analyse measurements of body composition made during the INSPIRE-22 expedition using the skin-fold method [20–22] and using DEXA measurements of body composition made pre- and post-expedition in the UK. Using the latter, changes in regional body composition as a result of undertaking the expedition were investigated for the first time. Skin-fold measurements are an indirect method of determining body composition [23] whereas in DEXA the amount of fat and fat-free tissue are quantified from images, albeit 2d images [24, 25]. Therefore, DEXA and skin-fold measurements of body composition made in the UK pre- and post-expedition were compared. Measurements of body fat using skin-fold thickness and body weight made during the INSPIRE-22 Antarctic expeditionary journey allow investigation of the assumptions behind using the CoE method to measure energy expenditure during the expedition.

## Materials and methods

In the Antarctic summer of 2022/2023, six men and three women participated in a 950km ski expedition from the Ronne Ice Shelf / land junction (altitude circa 200m) of the Antarctic continent to the South Pole (altitude 2840m) via the Messner Route hauling sledges weighing up to 85kg. The expedition lasted 47 days. This was the largest medical research expedition to date from the coast to the South Pole (https://www.inspire22.co.uk). The nine participants included six tri-service volunteers and three civilians. Participants were selected from British Army, Navy and Air Force volunteers and civilian volunteers with experience of extreme environments and/or expedition medicine following a year of selection and training activities. The final list of participants in the expedition was agreed 13^th^ November 2021.

### Study participants

The nine participants in the expedition, aged 28–63 years, were invited to participate in the scientific study of the impact of the expedition on their body composition, endocrinology, metabolism, and the functionality of wearable technologies. Participation in the scientific study was voluntary and independent of participation in the expedition. All nine participants volunteered to be participants in the scientific study and gave written informed consent prior to any data collection. Ethics approval for the study was obtained from the Ministry of Defence Research Ethics Committee (2125/MODREC/22) and the study was conducted in accordance with the Declaration of Helsinki. Unique participant identifiers were allocated in order of first study. Recruitment to the study started 1^st^ June 2022 and ended 24^th^ October 2022 when the final participant signed the consent form prior to the pre-expedition measurements at Coventry University.

### Data acquisition

#### Nutrition methodology

All participants created their own bespoke rations based on food products available from Antarctic Logistics and Expeditions (ALE), the logistics provider to the expedition (https://antarctic-logistics.com/). All participants created between five and seven different menus with most participants creating five menus with 10 days of each. The 50 days of rations was based on an anticipated expedition duration of 48 days with two days additional rations for emergencies.

An example menu is shown in S1 Table. Items included dehydrated breakfast and evening meal, hot drinks, sweet and savoury snacks to have during the day along with energy and recovery powders for drinks depending on participant preference. Participants were given a guideline on how much macro nutrients (carbohydrate, protein and fat) they should ingest each day based on recent guidelines for endurance athletes [26].

Participants entered calorific values for their selected food items into a spreadsheet giving detailed documentation of the macronutrient and calorific value of their nutrition on a day-by-day basis. A traffic light system was programmed into the spreadsheet so that participants could see if they were red, amber or green based on the guidelines [26].

Each participant had a log-book and at the same time every day during the expedition they recorded the percentage of the daily ration they had consumed. Any food swaps with other participants were recorded in this personal log-book. At the major resupply point (day 21) all participants recorded what food they had left over and gave this information to NT who recorded the information and shared it with HC, who was performing the nutrition analysis to ensure data were stored in two places to protect against data loss. These records included removing whole menus from a participants nutrition programme, food swapped between participants and extra food taken at the resupply.

On return to the UK, nutrition spreadsheets for each participant were reviewed in an interview session between HC and the participant where spreadsheet values were updated using contemporaneous notes held by both.

#### Body composition measurements

Immediately prior to departure from the UK and immediately after return to the UK whole body DEXA scans were performed on all participants to get body composition measurements. These measurements were made on a GE iDXA scanner. Within the two weeks prior to departure from the UK and within two weeks following return to the UK, participants attended Coventry University for a number of investigations including weight measurement and skin-fold measurements to the 7 site Jackson and Pollock protocol [20–22] performed by researchers certified to level-1 of the International Society for the Advancement of Kinanthropometry (ISAK). Skinfold measurements were made using Harpenden skinfold callipers (www.habdirect.com/product/harpenden-skinfold-caliper) and body weight measured using a SECA 704 column scales (www.seca.com).

One of the ISAK accredited researchers had previously trained two of the INSPIRE-22 participants in making the seven site Jackson and Pollock protocol skin-fold measurements and they made skin-fold measurements at five day intervals during the expedition using the same make and model of callipers. In addition to the skin-fold measurement, each participant’s weight was also measured using mechanical scales (Secca 874, www.seca.com). These measurements were made in the vestibule of the Hilleberg Kerron 4 GT expedition tents (https://hilleberg.com/eng/tent/black-label-tents/keron-4-gt/) where the internal dimensions were (l x w x h) 183 x 220 x 110cm max. A 100cm deep hole was dug in the snow in the vestibule so that the participant could stand without touching the roof of the tent. The weighing scales were placed on a level wooden board for the weight measurement. Measurements were made on rising and in the fasted state. Participants voided their bladder and wore standard clothing and footwear during the measurements.

**Fig 1:**
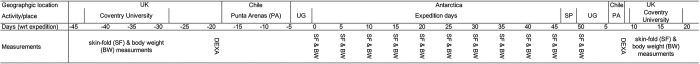
The geographical location and temporal sequence of body composition measurements. The timing of pre- and post-expedition measurements are made relative to the period of the expedition, taken as the period between the start of the expedition and return from the South Pole (SP) to the Union Glacier camp (UG). From the Union Glacier camp, participants travelled by air to Punta Arenas (PA) in Chile and from there, again by air, back to the United Kingdom (UK). Skin fold and body weight measurements are denoted by SF & BW and dual energy x-ray measurements of body composition by DEXA. It should be noted that the studies in Coventry took two days with participants studied in pairs wherever possible. The protocol for these studies included whole body calorimetry studies [1] and exercise studies the results of which are not reported in this paper.

The geographical location and temporal sequence of body composition measurements used in the study are shown diagrammatically in Fig 1.

#### Wearables

Throughout the expedition participants wore a GeneActiv activity sensor on the non-dominant wrist (https://activinsights.com). These devices have been previously shown to have a good correlation with energy expenditure determined by DLW during military training activities [27]. Each device contained a tri-axial accelerometer, a light detector and a temperature sensor. The expected maximum duration of the expedition was close to the anticipated battery life of the devices based on the manufacturer’s specifications and so each participant carried two devices. On day 34 of the expedition participants started wearing both devices, one on each wrist, and then on day 40 the first device was turned off and the second device transferred to the non-dominant arm. The second device, switched off, was carried on a sledge prior to being switched on and being worn; the first device was carried on a sledge after removal. The real-time clock of each of the GeneActiv devices used was set to local time before participants left Punta Arenas in South America and the sampling rate set to 10Hz.

### Data analysis

Images from the DEXA scanner were processed using software supplied by the scanner manufacturer together with the body weight measured as part of the scanning protocol to give a spreadsheet from each participant study with the total fat mass, total fat-free mass and the fat and fat-free mass for the following body segments: left and right arms, legs and trunk, plus the android and gynoid regions. The percentage of body fat, %fat, was calculated for each participant from both the pre- and post-expedition scans using:

$$\%fat=\frac{{fat}_{mass}}{fat_{mass}+{fat-free}_{mass}}$$ 1

Where *fat*_*mass*_ and *fat-free*_*mass*_ were the weights of the fat and fat-free tissue determined by the DEXA scanner’s software.

The percentage body fat was calculated from both the laboratory (UK) and expedition skin-fold measurements using the age related standard formula for men [20] and women [21]. The fat-free weight of each participant was then calculated as the difference between the total body weight and the fat weight. The change in these during the expedition for individual participants were compared graphically and changes correlated with the altitude at which the measurement was made.

The data from the GeneActiv devices were converted to comma-separated-variable format using the software supplied by the manufacturer, ActivInsights. From these data the acceleration in the x, y and z directions were extracted and a vector sum, SVM, calculated according to the formula in the GeneActiv documentation:

$$SVM=\sqrt{{A_{x}}^{2}+{A_{y}}^{2}+{A_{z}}^{2}}–1.0$$ 2

where *A*_x_, *A*_*y*_ and *A*_*z*_ are the are the accelerations in the x, y and z directions respectively in g and the -1.0 term is to remove the effect of gravity.

Energy expenditure measures during extreme activities are traditionally measured on a per-day basis and therefore the focus of our analysis is on time invariance of the daily activity. The 10Hz SVM data were resampled by selecting the maximum value for 120s non-overlapping time epochs throughout the data. During the expedition there were two rest days—days 21 and 31 –where the activity would clearly be different to the days when there was travel on the ice. Resampled SVM data from the remaining days were extracted where a day lasted 24 hours (1440 minutes) and was arbitrarily defined as starting at 08:00. Where data were available from both the first and second device, data up to day 37 was taken from the first device and data after day 37 was taken from the second device. Days when data were not available from either device were omitted from the analysis. These day data were concatenated in date order to form a time series of ‘activity for expedition travel days’. For individual subjects determining meaningful time averaged properties over a day requires the expedition time series for each day to be time invariant. For each participant, the mean values of the resampled SVM from each travel day in the expedition time series were determined together with the mean value from all expedition travel days. The distribution of values of the mean (first), second and third moments about the mean day for each participant were calculated. Both runs tests and χ^2^ tests were used to investigate whether the distribution of these values around the mean day was random. We want to be able to compare the daily energy expenditure of different participants so this requires the expedition time series between different participants to be statistically indistinguishable. The actigraphy data is not an absolute measure of activity so there will be a difference in absolute values between subjects. However relative patterns of activity during an expedition day should be the same for all subjects. To investigate this the mean day was calculated for each subject across all the expedition travel days and then normalized to the area under the curve, a measure of total activity for the day. Plots of the mean days for all subjects showed three were time shifted compared to the remaining six, suggesting an error in setting the real-time clock of the GeneActiv devices. The average of these six were determined and then the resultant cross-correlated with each of the other three to determine the time shift that needed to be applied to time-align them. Once all the average days from each participant were time-aligned, a χ^2^ goodness of fit between these and the mean day calculated across all participants was then performed. As a second part of ensuring time averaged measures can be compared between participants, autocorrelation functions were calculated from the expedition time series for each of the participants. Autocorrelation products for time-shifts of 0–1440 minutes (0-1day) were then normalized to the area under the curve and a χ^2^ goodness of fit between these and the mean day autocorrelation function calculated across subjects performed. The goodness of fit tests contained 720 points using the resampled SVM data. In applying the χ^2^ test to these data, the following should be noted:

- χ^2^ tests are sensitive to the values in them and therefore data sets were scaled so that the smallest values in the tests were > 3 before the test was applied [16, 28].
- the χ^2^ test had 717 degrees of freedom, above the number in standard statistical tables of limiting values, therefore the following formulae given in the statistical tables included in Bendat and Piersol [16] was used to determine the limiting value for significance at p < 0.05:

$$\chi_{n,\alpha }^{2}=n{\left[1-\frac{2}{9n}+z_{\alpha }\sqrt{\frac{2}{9n}}\right]}^{2}$$ 3

where *n* is the degrees of freedom and z_α_ is the percentage point for a standardised normal distribution.

#### Energy expenditure calculation

The energy expenditure during the expedition was calculated according to the following formula

$$EE=E_{nutr}+\frac{\Delta_{fat}{ED}_{fat}}{N_{days}}+\frac{\Delta_{fat-free}{ED}_{prot}}{N_{days}}$$ 4

Where *EE* is the average daily exergy expenditure during the measurement period of *N*_*days*_, *E*_*nutr*_ is average daily calorific intake, *Δ*_*fat*_ and *Δ*_*fat-free*_ are the change in weight of fat and fat-free tissues respectively, *ED*_*fat*_ is the energy density of fat and *ED*_*prot*_ the energy density of fat and protein. The third term of Eq 4 assumes that all fat-free tissue loss is muscle and so the energy density of protein is appropriate. Values of 9.461 kcal g^-1^ and 4.316 kcal g^-1^ were used for *ED*_*fat*_ and *ED*_*prot*_, respectively [3].

Quantification of nutrition was done for the periods 0–21 days and 22-47days. Measurements of body weights and skin-fold thickness, from which *Δ*_*fat*_ and *Δ*_*fat-free*_ weights are calculated were made every five days starting at day 0, the first day of the expedition. Since day 21 was a rest day the values at day 20 were taken for the start and end of the two periods for which nutritional intake was calculated, i.e. there was no weight or body composition changes on day 21, the rest day. The expedition reached the pole on day 47 and the values on day 47 were linearly extrapolated from the values at day 40 and day 45.

In order to understand the effect of using anthropometric data obtained during the expedition on determining the daily energy expenditure during the expedition, the energy expenditure was calculated from the weight measurements carried out at Coventry University pre- and post-expedition and from the weight measurements made at the start and end of the expedition This calculation was done assuming all weight loss was due to fat to avoid any differences being introduced as a result of different people making the skin-fold measurements to determine the percentage of fat.

Comparing results between male and female participants is problematical because there are only 3 of the latter. Therefore, where appropriate comparisons are made between individual values for the female participants with mean±standard deviation values for the male participants.

## Results

**Fig 2:**
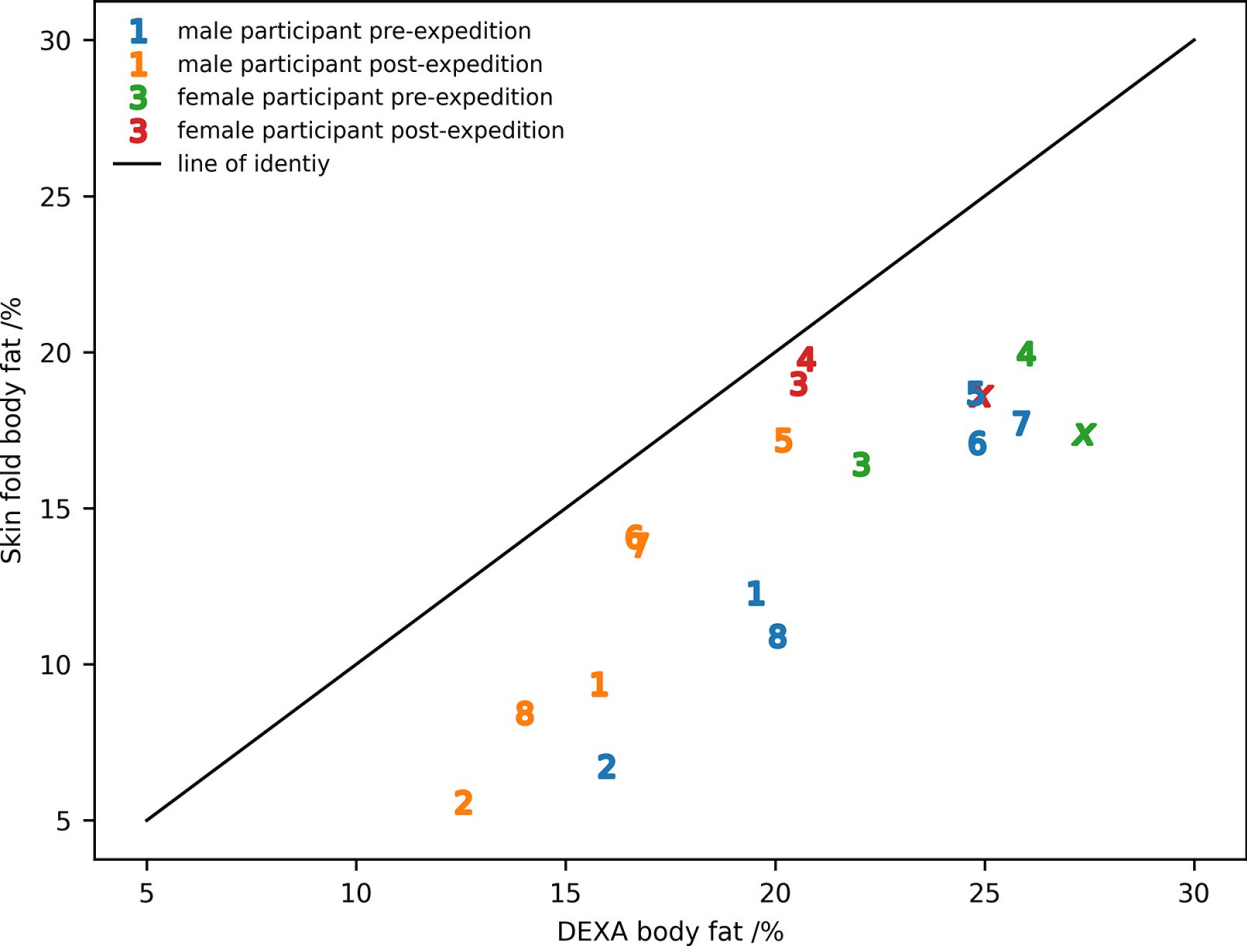
The comparison of body fat measured by DEXA and the skin-fold thickness for individual subjects pre- and post-expedition. Participants IS22001..IS22008 are identified by the numbers 1..8 and participant IS22010 by the roman numeral ‘x’.

The skin-fold method underestimated the percentage of body fat when compared with DEXA (Fig 2). The equation for the line-of-best fit between the skin-fold and DEXA measures for all values shown in Fig 2 was:

SF=0.86*D−2.97, 5

where SF and D are the percentage body fat determined by skin-fold measurements and DEXA, respectively. The fraction of the variance described by the line-of-best-fit, r^2^, was 0.69. It should be noted that the line-of-best-fit would have both a different slope and offset if the pre- and post-expedition measurements were considered separately. A Bland-Altman plot for the comparison is given in S1 Fig.

**Fig 3:**
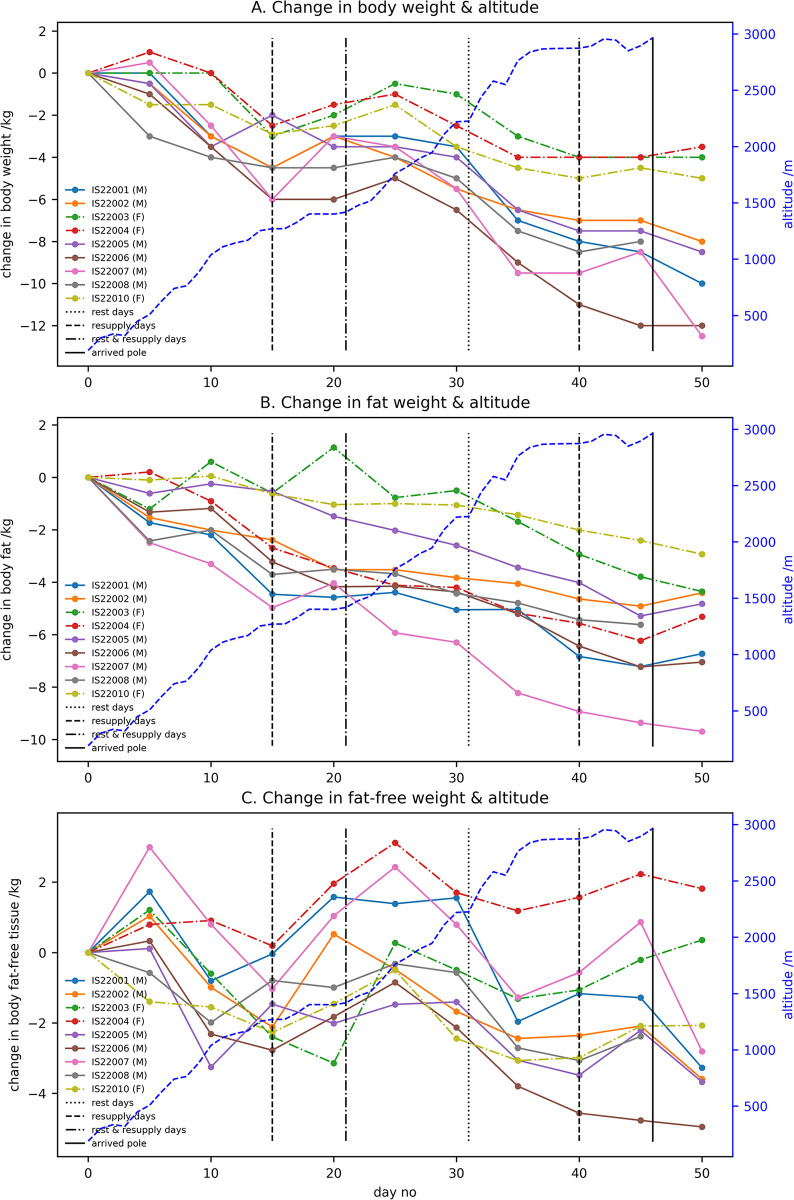
The change in body weight and body composition for individual participants during the expedition together with the altitude and key events during the expedition: A = body weight; B = fat weight; and C = fat-free weight. The altitude was measured daily whilst body composition measurements were made every five days and the dots show the days of measurement.

The change in body weight during the expedition is shown in Fig 3A together with the altitude and key events during the expedition. Most participants had an increase in body weight after the first re-supply point which also coincided with a reduction in the rate of ascent. The body weight peaked around 25 days for most participants and then slowly declined. It should be further noted that the start of this decline in body weight coincided with an increase in the rate of ascent and a slowing of the drop in body weight can be seen as the rate of ascent decreases at about 35 days as the expedition approached the pole. Visually, there are three distinct regions for the changes in body weight: up to day 20; after day 20 to day 30 and after day 30. For the male participants the mean (±standard deviation) for these three temporal regions are: -0.19±0.06; -0.12±0.10; and -0.24±0.10 kg/day respectively. For the three female participants the individual values are: (-0.10, -0.08,-0.13); (+0.10,-0.10,-0.10); and (-0.2, -0.1,-0.07) kg/day respectively. From these data, it is clear that all three female participants lost less weight after 30days than the average loss for the male participants. For the final measurements at 45 days just before reaching the pole the values are clustered based on sex with the exception of male subject IS22006, the oldest participant.

In addition to body weight, skin-fold thickness was also measured every five days during the expedition from which the percentage body fat and percentage fat-free tissue were calculated. The change in these from the start of the expedition are shown in Fig 3B and 3C. As before, these graphs also show the altitude and key events. The mean loss of fat weight for male participants was -0.15±0.04 kg/day and for the three female participants -0.08, -0.14 and -0.05 kg/day. For fat-free tissue the average loss for male participants was 0.04±0.04 kg/day whilst for female participants it was 0.00;0.05 and -0.05kg/day. With the exception of participant IS22003, Fig 3B shows a near linear decline in fat weight during the expedition, although the rate of fat loss is different for different participants. In contrast, Fig 3C shows that the fluctuations seen in the body weight (Fig 3A) are almost entirely due to changes in fat-free tissue weight.

Dividing the plots for fat (Fig 3B) and fat free tissue (Fig 3C) into the same three temporal periods used for body weight (up to day 20, days 20–30 and after day 30) the average rate of change in fat tissue for the male subjects for the three periods was -0.18±0.05, -0.09±0.08 and -0.15±0.06 kg/day, respectively whilst the loss for the three female subjects was (0.06, -0.17 and -0.05), (-0.16, -0.07 and 0.00) and (-0.22, 0.14 and -0.09) kg/day, respectively. Similarly, the average rate of change in fat-free-tissue for the male participants was -0.01±0.08; -0.03±0.08 and -0.09±0.08 kg/day whilst for the female participants it was (-0.16, 0.10, -0.070); (0.26, -0.03, -0.10) and (0.02, 0.04, 0.02) kg/day. From these data it can be seen that the major change is the reduction in the loss of fat-free tissue in female participants after 30 days.

Fig 3B and 3C show that there was a decrease in fat tissue between the start and end of the expedition but only a small change in fat-free tissue up to the expedition reaching the pole with a decrease in fat-free tissue in the final measurement made when the expedition was at Union Glacier camp (day 50). Quantifying this change by linearly extrapolating the weight at the pole from the measurements at 40 and 45 days gave a mean change in fat-free tissue for the male participants of -1.87±1.44 kg and of 0.39, -0.55 and -0.16kg for the individual female participants.

**Fig 4:**
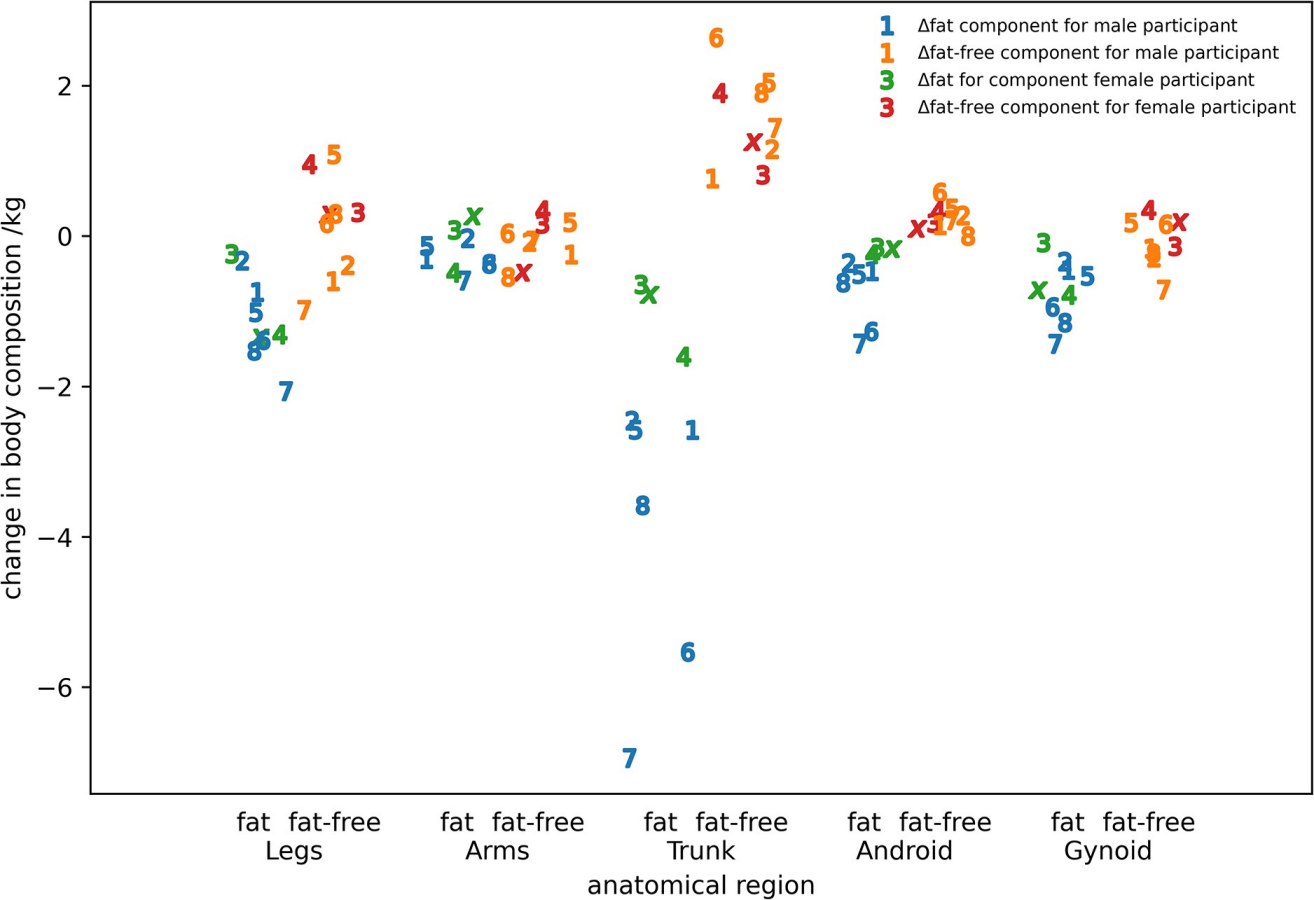
The change in composition for various body segments determined by DEXA scanning. Participants IS22001..IS22008 are identified by the numbers 1..8 and participant IS22010 by the roman numeral ‘x’.

Fig 4 shows the change in fat and fat-free tissue between the start and end of the expedition for different anatomical regions determined from the pre- and post-expedition DEXA scans. Whilst there was fat loss in most regions for the majority of participants, the largest fat loss was from the trunk. Whilst there was only a small difference in fat-free tissue weight in most regions, there was a clear fat-free tissue gain in the trunk.

**Fig 5:**
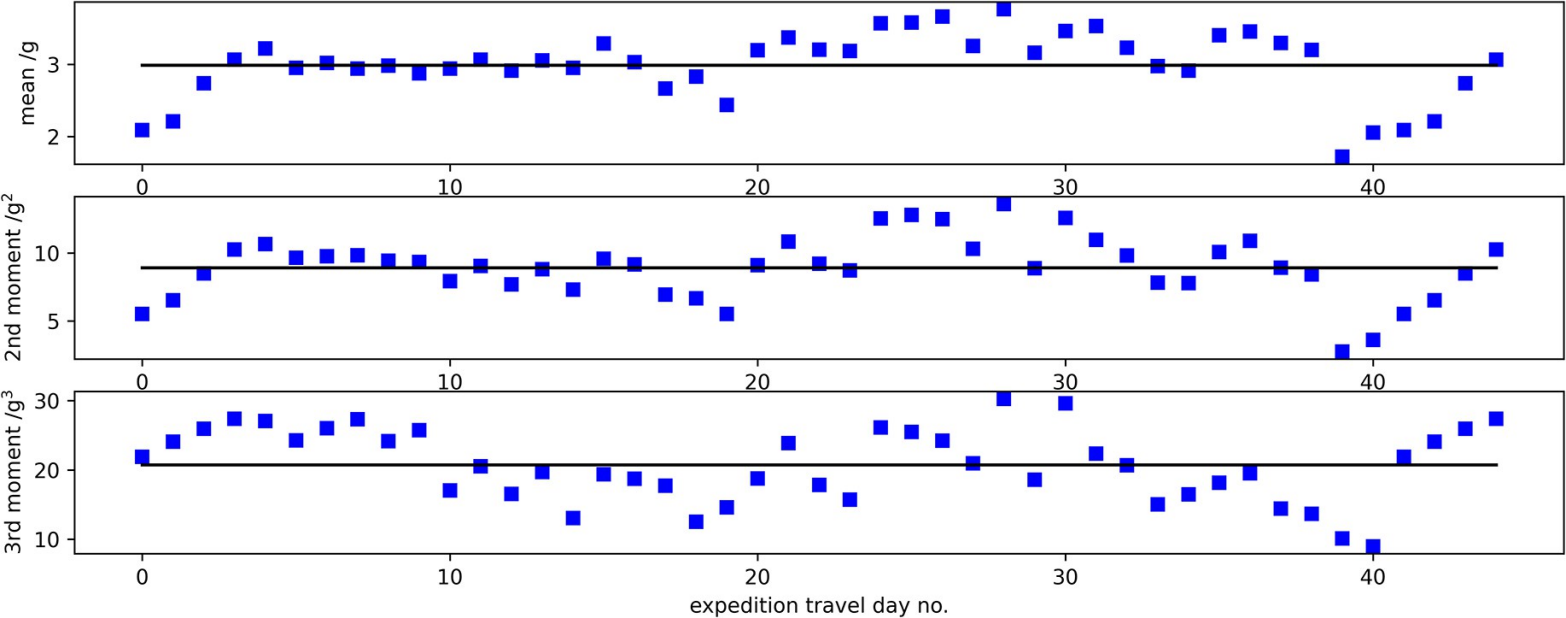
The day-by-day values of the mean activity measured by the GeneActiv accelerometers together with the mean value measured across all expedition travel days for participant IS22001.

Fig 5 shows an example plot of the mean, second and third moment expedition travel day time-series together with the mean values of each measure. The change in statistical properties between the start and the major resupply at 21 days, thereafter before and after the final ascent to the South Pole starting at expedition travel day 39 can be clearly seen; features that are common across participants. The expedition lasted 47 days and there were two rest days so the total number of expedition travel days was 45. Table 1 shows the number of expedition travel days on which data was available for each participant. Due to problems with the battery-life on the GeneActiv devices, complete data sets for all travels days were only available for two participants (IS22001 and IS22007) with two further participants achieving 44 days (IS22007 and IS22008) and one achieving 43 days (IS22003). The lowest number of days available was 27 days (IS22006). The data available from the GeneActiv devices for analysis from each participant are shown diagrammatically in S1 Fig.

**Table 1:** Results of the runs and χ^2^ tests on the ‘expedition travel days’ time-series.

Participant ID (sex)	Number of expedition travel days	Mean (1st moment)					2nd moment					3rd moment				
		no. days above measure mean	no. days below measure mean	runs	runs test p	χ2 test p	no. days above measure mean	no. days below measure mean	runs	runs test p	χ2 test p	no. days above measure mean	no. days below measure mean	runs	runs test p	χ2 test p
IS22001 (M)	45	25	20	16	0.03	>0.05	25	20	16	0.03	>0.05	22	23	9	0.00	>0.05
IS22002 (M)	34	19	15	8	0.00	>0.05	16	18	6	0.00	>0.05	19	15	12	0.04	>0.05
IS22003 (F)	43	27	16	9	0.00	>0.05	27	16	9	0.00	>0.05	28	15	16	0.12	>0.05
IS22004 (F)	40	22	18	18	0.36	>0.05	20	20	12	0.00	>0.05	18	22	20	0.80	>0.05
IS22005 (M)	34	19	15	13	0.09	>0.05	16	18	11	0.02	>0.05	14	20	15	0.37	>0.05
IS22006 (M)	27	17	10	7	0.01	>0.05	13	14	5	0.00	>0.05	13	14	5	0.00	>0.05
IS22007 (M)	45	27	18	18	0.15	>0.05	24	21	10	0.00	>0.05	19	26	9	0.00	>0.05
IS22008 (M)	44	25	19	16	0.04	>0.05	27	17	10	0.00	>0.05	22	22	14	0.01	>0.05
IS22010 (F)	44	21	23	13	0.00	>0.05	20	24	11	0.00	>0.05	22	22	25	0.54	>0.05

Applying a runs test to the data shown in Fig 5 from all participants gave p < 0.05 for all measures for all participants so the number of runs of values above and below the mean are not judged to be randomly distributed and therefore on the basis of this test the measures are not time invariant (Table 1). However, the χ^2^ test gave p > 0.05 for all measures and all participants so the daily values are deemed randomly distributed about the appropriate mean value so the data are time invariant on the basis of the χ^2^ test (Table 1).

**Fig 6:**
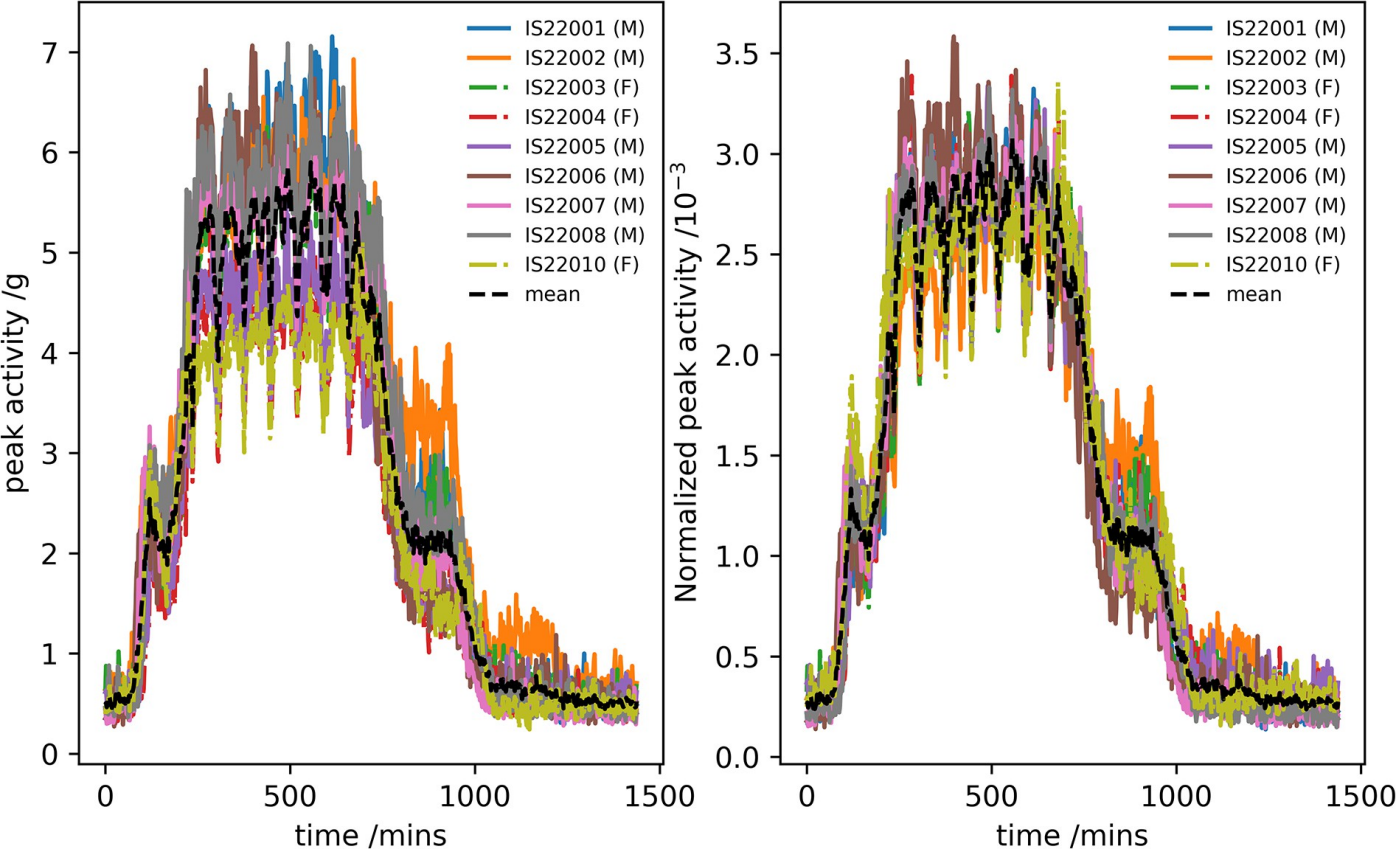
The mean day for each participant calculated from the expedition travel day time-series before (left) and after normalization (right) together with the mean value calculated across all participants.

Fig 6 shows the ‘average day’ activity data for the participants before and after normalization together with the mean measured across participants. The χ^2^ goodness-of-fit test applied to the normalized data showed no statistical difference (p > 0.05) between the individual participants and their mean.

**Fig 7:**
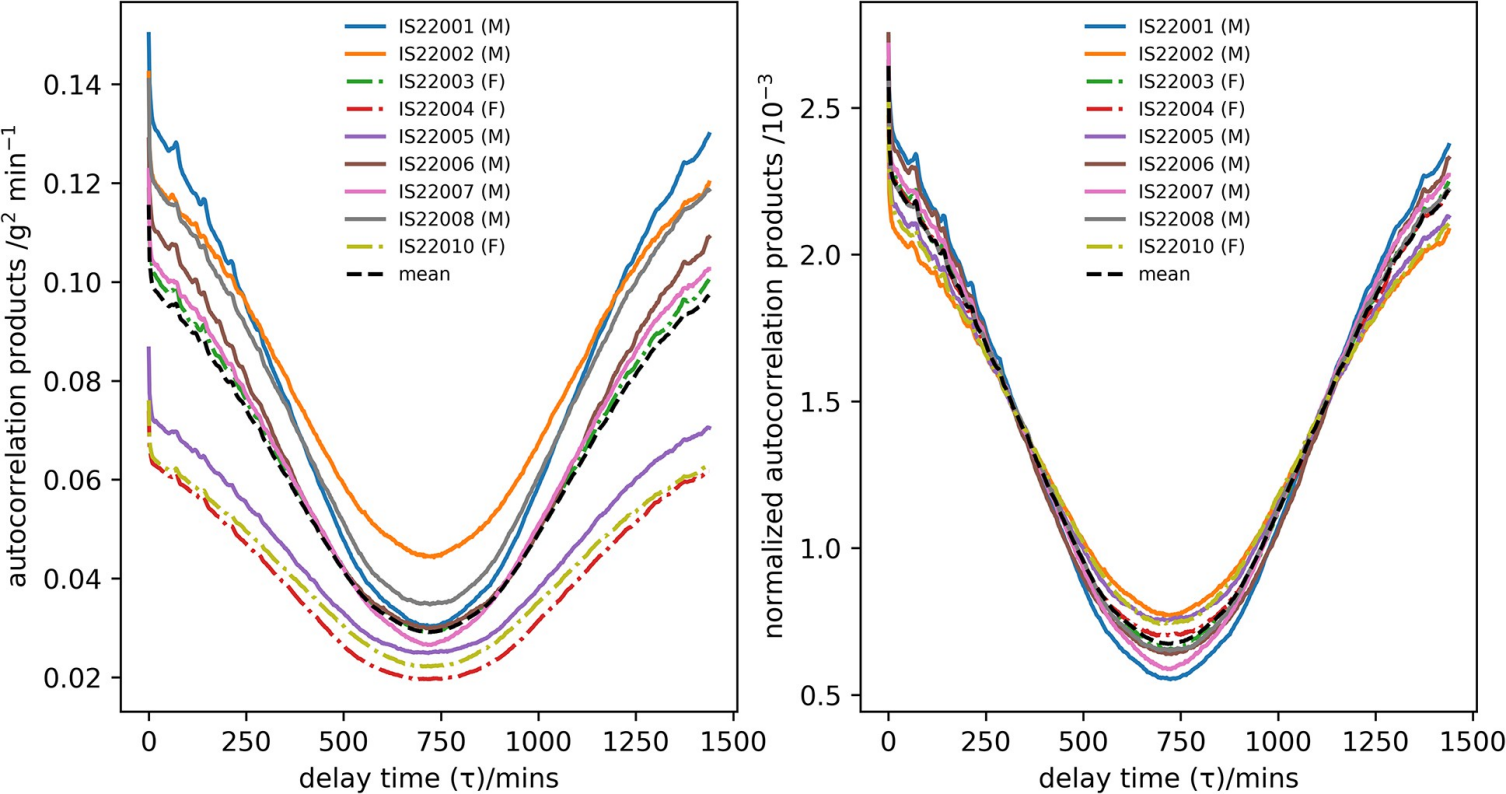
The autocorrelation function of the expedition travel days time-series data for each participant before (left) and after normalization (right) together with the mean value calculated across participants.

Fig 7 shows the autocorrelation function of the ‘expedition travel days’ time-series for the participants before and after normalization together with the mean measured across participants. The χ^2^ goodness-of-fit test applied to the normalized data showed no statistical difference between the individual participants and this mean (p > 0.05).

Table 2 shows the daily nutritional intake for participants up to the major resupply at day 21 and then from day 21 to the end of the expedition. There was a decrease in the daily calorific value consumed except for participants IS22004 and IS22005 where there was a small increase.

**Table 2:** The daily calorific intake through solids and fluids up to the major re-supply point at 21 days and thereafter in kcal.

Subject ID	0-21days	21-47days
IS22001 (M)	5164.40	4712.60
IS22002 (M)	5394.00	5307.50
IS22003 (F)	4528.40	3645.60
IS22004 (F)	3493.00	3649.80
IS22005 (M)	5748.40	5965.00
IS22006 (M)	5025.90	4571.10
IS22007 (M)	4720.10	3476.80
IS22008 (M)	5167.60	3942.20
IS22010 (F)	4605.70	4357.40
mean±sd	4871.9±647.2	4403.1±836.3

**Fig 8:**
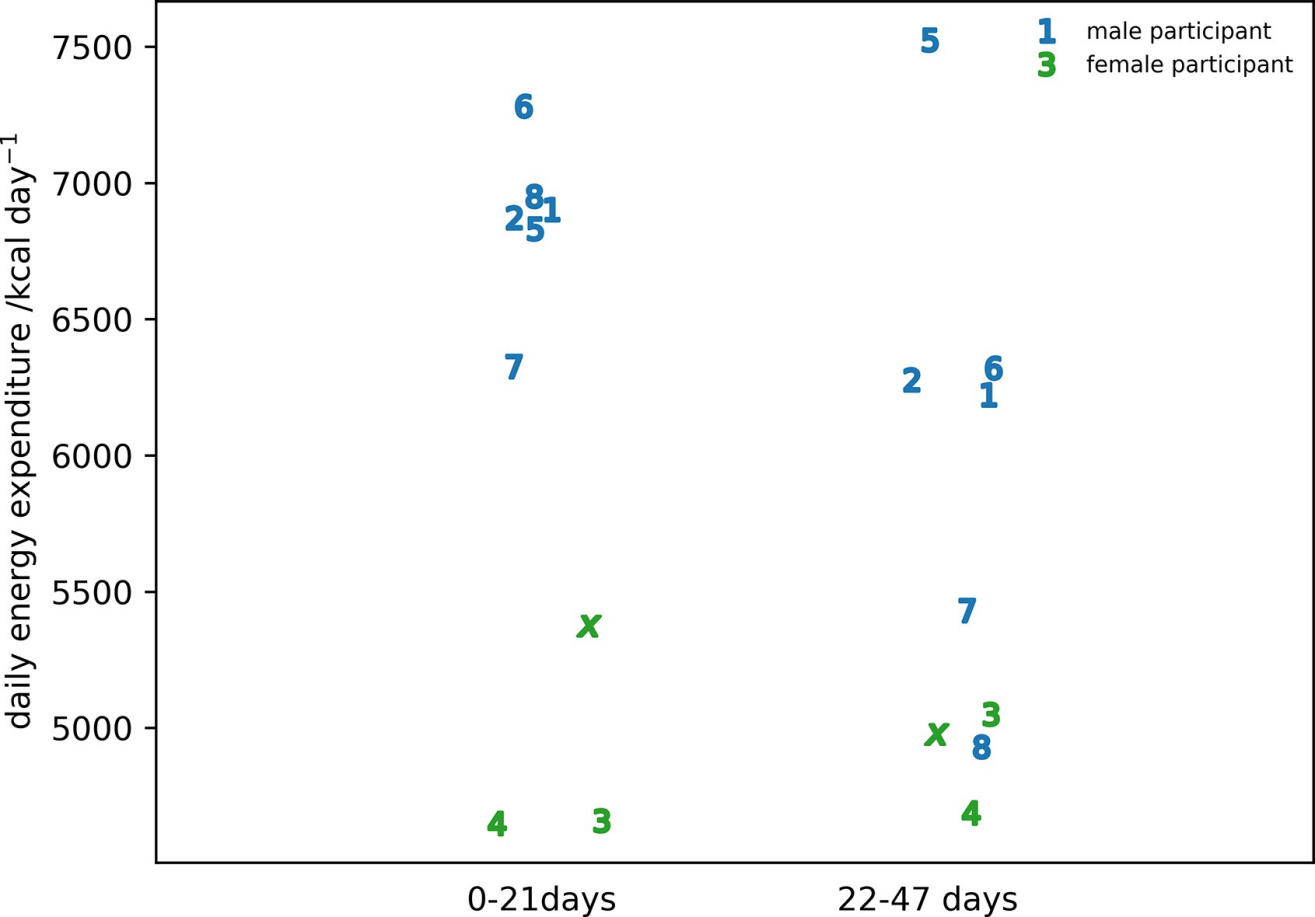
The daily energy expenditure calculated from the conversation of energy for the first 21 and second 25 days of the expedition. Participants IS22001..IS22008 are identified by the numbers 1..8 and participant IS22010 by the roman numeral ‘x’.

The results for calculating the total daily energy expenditure during the expedition using Eq 4 and the nutrition date from Table 2 are shown in Fig 8. For the majority of the male participants, the daily energy expenditure fell for the second part of the expedition compared to the first. One exception to this is participant IS22005 whose energy expenditure rose substantially between the first and second parts of the expedition. It should be further noted that this was the only male participant to increase their nutritional intake for the second part of the expedition (Table 2). The female participants showed a less homogeneous pattern with little or no change in two out of the three female participants but a substantial increase between the first and second parts of the expedition for IS22003.

The difference in body weight between the measurements made in the UK and the measurements made during the expedition are shown in Table 3. The difference in the mean weight between the beginning and the end of the expedition is 7.1kg whilst the difference between the pre- and post-expedition measurements made in the UK is 4kg.

**Table 3:** Body weight measurements made pre- and post-expedition in the UK and during the expedition itself together with the energy expenditure assuming the difference was due to change in fat weight. *It should be noted that the expedition ended on day 47 and the expedition end value was extrapolated from the measurements made on days 40 and 45.

Participant ID	UK measurements			Expedition measurements		
	Body weight [kg]		EE [kcal/day]	Body weight [kg]		EE [kcal/day]
	pre-expedition	post-expedition		expedition start	expedition end*	
IS22001 (M)	84.8	79.8	5911	87.0	78.3	6666
IS22002 (M)	75.9	75.4	5437	80.0	73.0	6755
IS22003 (F)	63.2	62.0	4282	63.0	59.0	4845
IS22004 (F)	65.2	63.1	4002	65.0	61.0	4385
IS22005 (M)	80.2	79.0	6110	85.5	78.0	7378
IS22006 (M)	83.3	76.1	6224	86.0	73.6	7270
IS22007 (M)	92.0	83.3	5784	91.5	83.4	5663
IS22008 (M)	85.0	78.8	5738	88.0	80.2	6060
IS22010 (F)	72.7	68.6	5294	71.5	67.2	5334
mean±sd	78.0±9.6	74.0±7.6	5420±784	79.7±10.6	72.6±8.6	6040±1062

Applying Eq 5 to both the pre- and post-expedition weight values and the start- and end-expedition weight values in Table 3 and the weighted average calorific intake in Table 2 gave values for total energy expenditure for these two sets of weight measurements (Table 3), assuming all weight loss is linear and entirely due to loss of fat. Whilst the difference in mean values is about 10% of the total energy expenditure, individual differences are much greater.

## Discussion

Our finding that the skin-fold thickness method underestimated body fat mass when compared to DEXA is consistent with the results reported in other studies that have presented results for healthy adult non-obese subjects [25, 29–33]. The correlation coefficient (r^2^) between percentage body fat determination by skin-fold thickness and by DEXA is in the middle of values from other studies where this is reported [25, 29, 30, 33]. Direct comparisons between studies must be treated with caution because of the different study methods used. The r^2^ value of 0.69 we obtained clearly indicates a substantial participant dependence on the correlation. In the context of this paper, making skin-fold measurements in a well-lit, warm laboratory with ample space is quite different to making measurements in the vestibule of an expedition tent in Antarctica when the air temperature inside the tent was typically -5 to +5°C. Whilst it was clearly impossible to perform DEXA scans during the expedition, it is reasonable to assume that the correlation would be at best the same as for the measurements made pre- and post-expedition, but more likely it could be substantially worse. Whilst the absolute values carry a high level of uncertainty, it is the relative values made during the course of the expedition that are primarily of interest in this paper. Whilst skin-fold measurements are not an ideal method of determining body fat, it is probably the only viable approach currently to obtaining body composition information throughout an expedition.

The values obtained for the overall change in body weight are consistent with the results previously reported for both men [1, 34, 35] and women [2]. However, our finding of substantial changes in the rate of change of body weight during the expedition was surprising. Where pre- and post-expedition measurements have been used previously to determine the energy expenditure during the expedition, there was always an assumption that weight loss was linear [1, 2]. Looking at the loss of fat and fat-free tissue, the linear loss of fat tissue was perhaps to be expected due to an increased basal metabolic rate due to the low temperatures [36] and the high levels of physical work undertaken every day pulling sledges weighing up to 85kg. Energy for this over and above that provided by the nutritional intake was coming from metabolising body fat to create glucose. The origin of the changes in fat-free tissue are much less clear. Previous work has shown that the change in fat weight is much greater than the fluctuations in fat-free weight [3] and our data show no substantial trend to participants losing fat-free tissue during the INSPIRE-22 expedition. Analysing regional changes in fat-free tissue from the DEXA scans performed in the UK we find minimal loss for all regions with the exception of the trunk where there is an increase in fat-free tissue in all participants. It is possible the fat-free tissue lost immediately after the end of the expedition and before the DEXA scans were performed came from the arms and legs when the high levels of physical activity ceased due to a cessation of sledge hauling. Changes in fat-free tissue during the expedition were occurring over short periods of time (less than five days which is the sampling interval) which suggests that water gain/loss could be occurring. Antarctica is the driest continent on earth and therefore insensible water loss will be substantial. However, this doesn’t explain the changes in fat-free weight. An altitude of 1,500m is reached on day 21 and a loss of up to 300ml of plasma volume has been reported at altitudes of 1,500–2,000m resulting in the increase in haemoglobin [37]. There is also evidence of the loss of fat-free tissue as the altitude increases followed by periods of recovery as the rate of climb decreases (Fig 4) and an explanation for this maybe a response to changes in physical workload.

Whilst the number of subjects is small, there appears to be a difference in the pattern of body mass and composition changes between male and female participants. Whilst the rate of loss of body mass for the three female participants was always less than their male counterparts making their body weight more stable, after 30 days the 3 female participants demonstrated preservation of fat-free mass compared with the 6 male participants. These observations are consistent with known sex differences in substrate utilisation during an energy deficit, with women using relatively more fat and less carbohydrate or protein than men [38], contributing to better preservation of fat-free mass among women [39]. The female energy economy may be better adapted to extreme endurance events like a polar ski traverse or multi-stage ultramarathon, perhaps helping to account for the reduced difference with sex in performance recently observed with greater duration of events, noting that a multiplicity of other factors are also likely to contribute to any difference [40].

The number of participants on whom a complete or near complete set of actigraphy data were available was disappointing. Care was taken to use two devices and the way they were stored and used during the expedition followed discussions with the manufacturers. All devices used were new and purchased specifically for the expedition. The memory, other electronic components and the battery within the devices have a contra-indication for use in the temperatures encountered in Antarctica (down to -50°C). The manufacturer specifications for the device pre-expedition did not include a minimum operating temperature but most commercial electronic devices will only work down to 0°C and many manufacturers of portable electronic devices (e.g. mobile phones) specify a minimum operating temperature of 5°C. Data in all devices were readable on return to the UK and therefore the problem was probably batteries losing charge on exposure to cold temperatures, albeit possibly for only short periods of time. The procedures for using the GeneActiv devices need to be re-examined ahead of their use on another Arctic or Antarctic expedition. Interestingly, recent specifications for the latest version of the device available just before submitting this paper (April 2024) have included a working temperature range of 5-35°C and have reduced the expected maximum storage time based on battery life from 60 days to 44 days. These values would fit well with our experience.

Time invariance of data during the expedition is an essential pre-requisite for obtaining meaningful time-averaged quantities for energy expenditure. In this context, it is important that there is little day-to-day variation in the activity levels of individual subjects. The runs test is the only commonly used statistical test that takes into account the time history of the data and is the gold-standard test for stationarity (time-invariance) of random data [16] where the runs test is investigating whether there are trends in the data. However, the expedition accelerometery data are not random data but rather the activity on a day-by-day basis that is dependent on the weather, the ice-conditions and the change in altitude. In addition, whilst the relationship between accelerometery and activity is assumed to be monotonic, it is almost certainly non-linear for activities in the polar environment. Therefore, the requirement that runs of day-to-day values above and below the mean are randomly distributed is considered unrealistic on the basis of the data presented in this paper. For example, Fig 2 shows a clear difference in statistical properties of the mean and moment measures up to and after the resupply at 21 days and then again when the participant undertakes the final climb from the polar plateau to the pole on travel day 39 (expedition day 41). However, the χ^2^ test shows that the distribution of values for each measure above and below their mean value are randomly distributed supporting the view that overall the day-by-day data is time invariant. In order to make comparisons between subjects, the day-to-day values from the different participants should be statistically indistinguishable from the mean. As accelerometery is not a direct measure of activity and the relationship between the two will be different for different individuals, the measures to test time invariance need to be normalised as part of the analysis. The criteria for stationarity across multiple records in random data is that the mean and autocorrelation function of individual records are indistinguishable from their mean values for all participants. The χ^2^ goodness-of-fit test applied to the normalized mean day and autocorrelation function data show that there is day-by-day time invariance across the participants. Polar expeditionary travel may be unique in providing this. Mountaineering expeditions will tend to present increasing challenges, both climbing and environmental, during the ascent and other extreme expeditionary ventures, particularly competitive ones, will not have time-invariance in activity across subjects. For example, in oceanic rowing, where different approaches are used to gain competitive advantage [9], data will not be time invariant on a day-to-day basis across subjects.

Nutrition for the expedition was selected by the participants with a recommendation that the calorific values and macronutrient content be kept within agreed guidelines. This contrasts with other recent expeditions where nutrition was designed for individuals [2]. Changes during the expedition between participants, particularly before the first major resupply at 21 days, were due to personal preference. The reduction in the nutrition consumed by most participants after 21 days was not the result of food shortage but rather from satiety. The impact of this reduction can only be understood by considering the energy expenditure.

It should be noted that the daily energy expenditure was calculated as the average over 2 consecutive periods: the first being 21 days; and the second being 26 days where each period included 1 rest-day. However, as the number of travel days is much greater than the number of rest days and the energy expenditure for Antarctic travel is much greater than the basal metabolic rate, the errors will be small. Skin-fold measurement of percentage body fat on these participants underestimated the percentage of body fat against DEXA. Assuming DEXA is the gold-standard, then the values used to determine energy expenditure underestimate the changes in fat weight and over-estimate the changes in the weight of fat-free tissue giving an overall under-estimate of energy expenditure since the energy density of fat is higher than the energy density of protein. Values of energy expenditure during the expedition are in line with those found previously for both men, [3, 34, 35] and women [3]. It is interesting to note that the highest values during both the first and second period of calculation are from the two oldest male members of the expedition which is in line with what has been reported previously [1, 7, 8]. The reason for the substantial increase in energy expenditure for participant IS22005 after 21 days is unexplained. The modest loss of body weight shows that the nutrition was appropriate for the activity. Exposure to altitude greater than 1500m occurs after 21 days and, perhaps surprisingly, this coincides with a drop in energy expenditure. The rate of climb before and after 21 days are similar although the temperature decreases with increasing altitude would suggest there should be an increase in basal metabolic rate to maintain body temperature. A 60% increase in basal metabolic rate on exposure to the Antarctic environment has been reported [36]. However, clothing was appropriate to the environment with only the face exposed. Previous work has shown that cooling of the face does not increase basal metabolic rate [41]. Acclimatization must also be considered as a possible explanation for the difference in energy expenditure between the first and second parts of the expedition. The time period over which this happens is dependent on the activation of brown fat tissue which occurs, if present, after about 10 days exposure to cold [42]. Making the reasonable assumption that the nutritional input during the first 21 days of the expedition was approximately constant then loss of fat can be used as a proxy for change in energy expenditure (Fig 4). There is little evidence from Fig 4 of a substantial change in energy expenditure for the first and second 10-day periods of the expedition.

To look at the effect of using anthropometric measurements during the expedition rather than the pre- and post-expedition measurements made in the UK, the change in weight for the two sets of measurements and the corresponding daily energy expenditure were determined. The difference in mean weight between showed participants had regained about 60% of their lost body weight between the end of the expedition and measurements being made in the UK. Therefore, a loss of muscle mass due to a cessation of the sustained strenuous activity of pulling heavy sledges but a gain in weight due to uncontrolled access to palatable nutrition is likely. Our data show skin-fold thickness measured by accredited and expert researchers underestimating body fat determined by DEXA. Measurements during the expedition were made by participants and the relationship to DEXA (or other methods) of measuring body composition is unknown. Therefore, the energy expenditure in this part of the analysis assumed the weight loss was due to fat loss; an assumption that has been made in the past [1]. Daily energy expenditure determined from the UK weight measurements was about 90% that determined from weight measurements made during the expedition. The smaller percentage error is because the weight loss is modest and therefore nutrition makes up about 87% of the energy expenditure in our study population. For larger weight losses, for example the 25% loss of body weight reported in the paper on one expedition [8], the difference between measurements made during the expedition and measurements made in the UK would be greater.

One of the aims of studying physiology in extreme environments (sometimes termed ‘extreme-medicine’) is to achieve translation to clinical medicine [e.g. 43]. The time-invariance of physiological and anthropometric measures from polar expeditions provides an opportunity, perhaps a unique opportunity, to understand the physiological consequences of long-term disease processes and their treatment regimens including cancer, diabetes and obesity.

## Supporting information

S1 Table An example planned menu for 1 day taken from the spreadsheets used to select food pre-expedition. The dried fruit and nuts (items 10,11) and crackers etc (items 12, 16, 17 &19) were in the graze bags and the amount consumed quantified as part of the nutritional monitoring. Protein drinks and soups (items 13, 15) were consumed at the end of each day following establishing camp and before dinner. The butter (item 18), if used, was melted into the dinner pouch. Once again, the amount of these items actually consumed was determined as part of the nutritional monitoring. Note: weight values have been rounded to the nearest gram.
(DOCX)

S1 Fig A diagram showing the data availability from the GeneActiv devices used in the study. (JPG)

S2 Fig A Bland-Altman plot for DEXA and skin-folds fat measurements. Participants IS22001..IS22008 are identified by the numbers 1..8 and participant IS22010 by the roman numeral ‘x’.
(TIF)
