# Supplementary material for: Changes in body composition and average daily energy expenditure of men and women during arduous extended polar travel
Source: PLoS One. 2024 Oct 10;19(10):e0308804. doi: 10.1371/journal.pone.0308804 (PMC11466405; doi:10.1371/journal.pone.0308804)
Supplement: S1 Table — The dried fruit and nuts (items 10,11) and crackers etc (items 12, 16, 17 &19) were in the graze bags and the amount consumed quantified as part of the nutritional monitoring. Protein drinks and soups (items 13, 15) were consumed at the end of each day following establishing camp and before dinner. The butter (item 18), if used, was melted into the dinner pouch. Once again, the amount of these items actually consumed was determined as part of the nutritional monitoring. Note: weight values have been rounded to the nearest gram. (DOCX) [file pone.0308804.s001.docx]

| Serial | Meal | Brand | Food | kcals | Weight /g | | | |
| --- | --- | --- | --- | --- | --- | --- | --- | --- |
|  |  |  |  |  | Portion | Carbs | Fat | Protein |
| 1 | Breakfast | Firepot | Baked apple porridge | 660 | 165 | 140 | 11 | 15 |
| 2 | Dinner | Firepot | Chicken Keema curry | 835 | 190 | 103 | 14 | 64 |
| 3 | Lunch | Pro Peak snack | Sea Salt caramel | 150 | 37 | 23 | 6 | 4 |
| 4 | Lunch | Gu Stroopwaffel | Hot chocolate | 150 | 32 | 22 | 6 | 1 |
| 5 | Lunch | Gu Energy Gel | Mint choc | 100 | 32 | 22 | 2 | 0 |
| 6 | Lunch | Pro bar bolt | energy chew | 190 | 30 | 46 | 0 | 0 |
| 7 | Lunch | Pro bar meal | superberry and greens | 370 | 85 | 50 | 16 | 9 |
| 8 | Lunch | Pro bar meal | chocolate coconut | 390 | 85 | 50 | 18 | 8 |
| 8 | Drinks | Mike and Jens | Hot chocolate | 280 | 64 | 46 | 8 | 6 |
| 9 | Lunch | Ritter Sport | Average Chocolate | 554 | 100 | 47 | 36 | 7 |
| 10 | Lunch |  | Dried Fruit | 186 | 60 | 48 | 0 | 1 |
| 11 | Lunch |  | Mixed Nuts | 361 | 60 | 12 | 32 | 13 |
| 12 | Lunch | Kryzpo | Crisps (1/3 of a container) | 219 | 43 | 25 | 12 | 3 |
| 13 | Lunch |  | Soup | 58 | 15 | 11 | 1 | 1 |
| 14 | Drinks |  | Accelerade | 120 | 31 | 20 | 1 | 5 |
| 15 | Drinks |  | Protein Drink | 270 | 74 | 52 | 2 | 13 |
| 16 | Lunch |  | Crackers | 189 | 46 | 28 | 6 | 5 |
| 17 | Lunch |  | Cheese | 256 | 71 | 7 | 19 | 16 |
| 18 | Dinner |  | Butter | 145 | 20 | 0 | 16 | 0 |
| 19 | Lunch |  | Salami | 196 | 50 | 0 | 17 | 11 |
| Total | | | | 5679 | 1290 | 752 | 223 | 182 |
