# Supplementary figures and images for: Changes in body composition and average daily energy expenditure of men and women during arduous extended polar travel
Source: PLoS One. 2024 Oct 10;19(10):e0308804. doi: 10.1371/journal.pone.0308804 (PMC11466405; doi:10.1371/journal.pone.0308804)

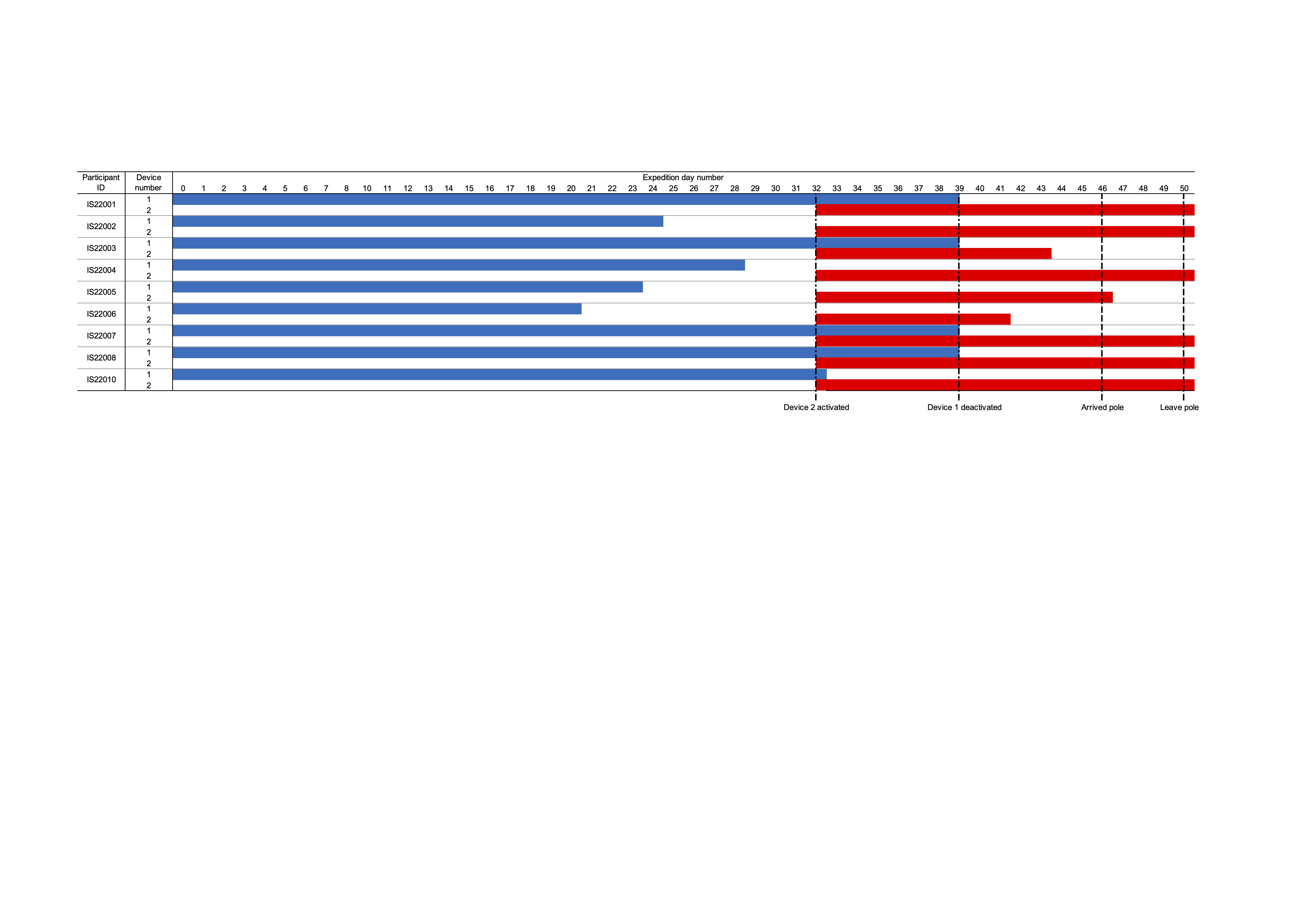

Supplement: S1 Fig — (JPG) [file pone.0308804.s002.jpg]

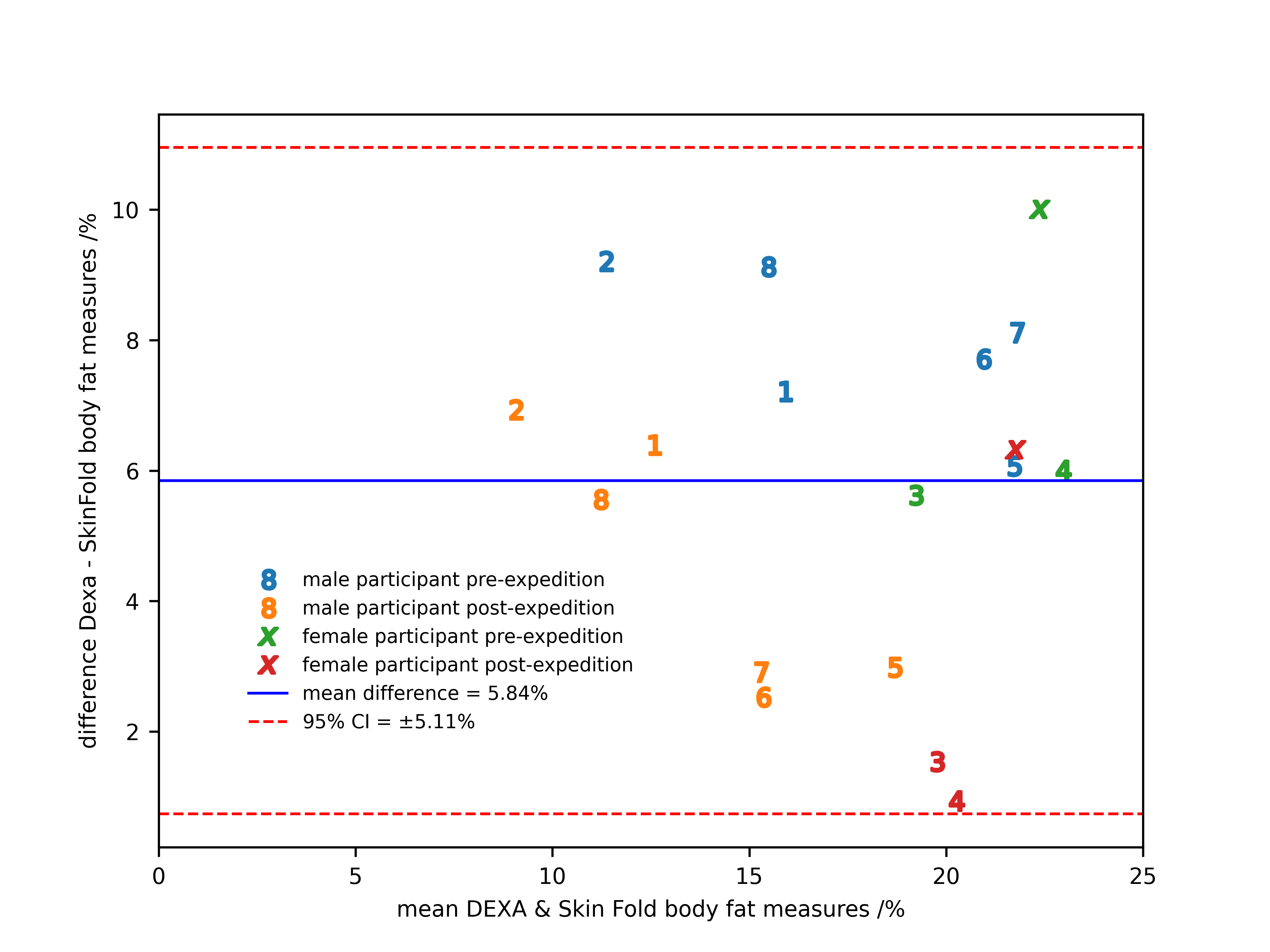

Supplement: S2 Fig — Participants IS22001..IS22008 are identified by the numbers 1..8 and participant IS22010 by the roman numeral ‘x’. (TIF) [file pone.0308804.s003.tif]
